# The inclusion of real world evidence in clinical development planning

**DOI:** 10.1186/s13063-018-2769-2

**Published:** 2018-08-29

**Authors:** Reynaldo Martina, David Jenkins, Sylwia Bujkiewicz, Pascale Dequen, Keith Abrams, on behalf of GetReal Workpackage 1

**Affiliations:** 1 Department of Health Sciences, University of Leicester, University Road, Leicester, UK; 2 Department of Biostatistics, University of Liverpool, 1-5 Brownlow Street, Liverpool, UK; 3 School of Health Sciences, University of Manchester, Oxford Road, Manchester, UK; 4 Evidence Synthesis/Health Economics, Visible Analytics Ltd., Union Way, Oxon, UK

**Keywords:** Network meta-analysis, Relapse rate, Negative binomial model, Clinical development plan, Sample size, Clinical trial simulation

## Abstract

**Background:**

When designing studies it is common to search the literature to investigate variability estimates to use in sample size calculations. Proprietary data of previously designed trials in a particular indication are also used to obtain estimates of variability. Estimates of treatment effects are typically obtained from randomised controlled clinical trials (RCTs). Based on the observed estimates of treatment effect, variability and the minimum clinical relevant difference to detect, the sample size for a subsequent trial is estimated. However, data from real world evidence (RWE) studies, such as observational studies and other interventional studies in patients in routine clinical practice, are not widely used in a systematic manner when designing studies. In this paper, we propose a framework for inclusion of RWE in planning of a clinical development programme.

**Methods:**

In our proposed approach, all evidence, from both RCTs and RWE (i.e. from studies in routine clinical practice), available at the time of designing of a new clinical trial is combined in a Bayesian network meta-analysis (NMA). The results can be used to inform the design of the next clinical trial in the programme. The NMA was performed at key milestones, such as at the end of the phase II trial and prior to the design of key phase III studies. To illustrate the methods, we designed an alternative clinical development programme in multiple sclerosis using RWE through clinical trial simulations.

**Results:**

Inclusion of RWE in the NMA and the resulting trial simulations demonstrated that 284 patients per arm were needed to achieve 90% power to detect effects of predetermined size in the TRANSFORMS study. For the FREEDOMS and FREEDOMS II clinical trials, 189 patients per arm were required. Overall there was a reduction in sample size of at least 40% across the three phase III studies, which translated to a time savings of at least 6 months for the undertaking of the fingolimod phase III programme.

**Conclusion:**

The use of RWE resulted in a reduced sample size of the pivotal phase III studies, which led to substantial time savings compared to the approach of sample size calculations without RWE.

## Background

The drug development process is generally divided into different phases. Trials up to phase II are considered exploratory. Phase III trials are generally designed based on the results of the phase II trials. Some design elements of the phase III trials occasionally also include evidence from other relevant randomised controlled trials (RCTs). A recent survey among participants of an International Clinical Trials Methodology Conference confirmed that evidence synthesis is not routinely used in the design and analysis of clinical trials [1]. The use of real world evidence (RWE), such as that from observational and pragmatic studies, in the evaluation of new health technologies and in clinical development programmes has recently become a prominent topic of an international debate [2]. Sutton et al. [3] provided a framework for the synthesis of available evidence with the aim of maximising the use of relevant information from existing data sources and thus reducing the need for future studies. Reducing the need for, or at least the size of, future studies has a significant impact on the cost of drug development, and potentially this can be achieved if, for example, the synthesis of available evidence results in more precise estimates of effectiveness. The use of RWE is currently being explored as the way toward bridging the effectiveness-efficacy gap [4, 5], i.e. the difference between the efficacy observed in RCTs and the more general effectiveness observed in trials conducted under general practice (real world conditions). To our knowledge, RWE has not been widely included in drug development programmes, for example, to inform future (phase III) studies. However, there have been a few published examples of the use of meta-analysis to inform sample size [6] and the design of future trials [7–9].

The use of only phase II data to design phase III trials has been met with criticism due to the nature of the phase II trials used, which are generally exploratory in nature, and the lack of reproducibility of sample sizes and clinical results of the phase III trials. For example, Tomblyn and Rizzo [10] reported that the use of phase II trial data can be misguided in clinical practice due to the exploratory nature of the trials. Zia et al. [11] compared outcomes of phase II studies with subsequent randomised controlled studies using identical chemotherapeutic regimens. It was reported that 85% of the phase III studies had lower response rates than those of the preceding phase II trial and the response rates were on average about 12.9% lower in the phase III studies compared to those of phase II. The lower response rates indicate that these phase III studies were not sufficiently powered. The issues of underpowered studies and poor assumptions in sample size calculations were also reported in the work of Vickers [12] and Charles et al. [13].

De Ridder [14] did not limit the design of a phase III trial to the use of the reported phase II data, but performed modelling and simulation of the phase II data to investigate key design issues such as sample size, the doses or trial duration. DeSantis and Zhu [15] undertook a mixed treatment comparisons meta-analysis and used the results to inform the power and design of a future hypothetical trial. Cameron et al. [16] provided an overview of the challenges and opportunities to include randomised and non-randomised clinical trials in a network meta-analysis for assessing the safety and effectiveness of medical treatments.

The aim of this paper is to describe a novel strategy for inclusion of all available RCT data and RWE in a step-wise approach. By performing network meta-analyses (NMAs) at key stages of development, informed decisions can be made based on all available data. We aim to synthesise the available evidence from phase II and phase III RCTs, including drug trials in real world conditions, and illustrate how the obtained information can be applied to design phase III drug trials in a clinical development programme.

## Methods

The following sections describe the strategy for including RWE in the drug development process in a step-wise and recursive approach. The proposed method is an extension of the approach taken by DeSantis and Zhu [15] and Sutton et al. [6], who claimed that basing the sample size of a new trial on an updated meta-analysis may make more sense than powering the trial in isolation.

We consider the sequential implementation of NMAs that include RCTs and RWE available at key stages of development. The methods are illustrated using an example development for relapsing remitting multiple sclerosis (RRMS).

The annualised relapse rate (ARR) was the primary variable in the development programme and RWE studies obtained. This variable is used throughout this manuscript to illustrate the proposed strategy.

### Strategy for the inclusion of RWE in the drug development process

The procedure performed to include RWE at key stages of development can be summarised in the following steps:

- Step 1
  - Use NMA (to include phase II trial [17] and all RCT and RWE available prior to the design and execution of the TRANSFORMS trial [18]) to obtain estimates of relative effectiveness.
  - Perform NMAs sequentially in time to include each phase III trial separately in the meta-analysis of all earlier studies, and then finally include all RCTs together.
  - Extract ARR and standard error from the NMA to use in simulation of alternative development strategies.
- Step 2
  - Use estimates from the NMA based on the predictive distribution [19] to simulate the effects and confidence interval of a future alternative phase III trial (by sampling from a negative binomial distribution).
  - Assess how many patients will be needed to execute a similar phase III trial with sufficient power (90%), whilst the probability of falsely claiming effectiveness remains low (< 5%).
- Step 3
  - Compare the size of the original trial with the size of the simulated trial.
- Step 4
  - Investigate whether the original trial could have been executed successfully with the alternative number of patients by analysing the original trial with a lower number of patents.
  - Using the (lower) number of patients obtained from the simulated trials, run clinical trial simulations assuming the treatment effects are as observed in the reported clinical trials.
  - Assess power and false positive rate.
- Step 5
  - Include the size and relative effect estimates of simulated trials instead of the original trial results in an NMA to investigate whether or not there is a difference in the totality of evidence of effectiveness between the original NMA and the NMA based on simulated results.

Steps 2 through 4 are repeated for each phase III trial.

The following section describes the example development used to illustrate the proposed strategy. The sections titled Network meta-analysis methods and Clinical trial simulations describe the NMA and the clinical trial simulations. The applied software is summarised in the section titled Software.

### Illustrative example

This work has been undertaken as part of the Innovative Medicines Initiative (IMI). As part of this initiative, RCT information was made available by a sponsor to illustrate the methodological proposals described in this manuscript. Hence, the development studies of fingolimod have been used in this illustrative example. The sequential NMA was designed to include RWE available after the execution of the phase II trial of fingolimod [17] and prior to the design and execution of the TRANSFORMS [18], FREEDOMS [20] AND FREEDOMS II [21] phase III RCTs.

The primary variable in the phase III trials was the ARR.

A brief description of the pivotal studies in this illustrative development programme is provided in Table 1. The table includes the study reference, treatments in each study, number of patients in each study, primary and key secondary outcomes and whether or not the power and/or statistical significance level was included in the publication. The information included in the table was obtained through a literature search.

**Table 1:** Summary of pivotal trials in the illustrative development plan

Study	Treatments	No. of patients (*N*)	Primary outcome	Relevant secondary outcomes	Power/significance level
Phase II [17]	Placebo or fingolimod 1.25 mg or fingolimod 5 mg	281	Hamburg Quality of Life Questionnaire (HAQUAMS) and Beck Depression Inventory second edition (BDI-II)	Not published	Not published
TRANSFORMS [18]	Interferon beta-1a or fingolimod 0.5 mg or fingolimod 1.25 mg	1292	Annualised relapse rate	Change in Expanded Disabiity Status Scale (EDSS) score	90%/5% (two-sided)
FREEDOMS [20]	Placebo or fingolimod 0.5 mg or fingolimod 1.25 mg	1272	Annualised relapse rate	Change in EDSS score	Power not published/5% (two-sided)
FREEDOMS II [21]	Placebo or fingolimod 0.5 mg or fingolimod 1.25 mg	1083	Annualised relapse rate	Change in EDSS score	90%/5% (two-sided)

Table 2 summarises the RWE studies included in the NMA, the treatments, the number of subjects, number of relapses, the person years and the Expanded Disability Status Scale (EDSS). The studies were obtained through a literature search. The ARR is estimated by dividing the number of relapses by the person years.

**Table 2:** RWE studies included in the NMA and main data extracted per treatment

Study	Treatments	No. of subjects (*N*)	No. of Relapses	Person years	EDSS
Lanzillo (2012) [39]	Natalizumab	42	10	42	3.5
	Rebif 44	42	23	42	
Limmroth (2007) [40]	Avonex	1094	1116	2188	2.62
	Betaferon	1034	1075	2068	
	Rebif 22	555	588	1110	
	Rebif 44	185	233	370	
Halpern (2011) [41]	Natalizumab	288	21	72	
	Avonex	151	7	38	
	Rebif 22	329	22	82	
	Betaferon	144	11	36	
	Glatiramer acetate	469	25	117	
Patti (2006) [42]	Betaferon	114	137	570	2.2
	Avonex	37	50	185	
	Rebif 22	17	35	85	
Río (2005) [43]	Placebo	107	288	356	2.73
	Glatiramer acetate	101	204	334	
Haas and Firzlaff (2005) [44]	Avonex	79	109	158	2.2
	Betaferon	77	123	154	2.28
	Glatiramer acetate	79	56	158	1.98
		48	59	96	2.36
Khan et al. (2001) [45]	Placebo	15	23	23	2.63
	Avonex	34	41	51	2.71
	Betaferon	34	28	51	2.6
	Glatiramer acetate	39	29	59	2.64
Trojano et al. (2003) [46]	Betaferon	209	136	418	2.5
	Avonex	169	120	338	2.4
Carra et al. (2003) [47]	Avonex	26	14	35	2.02
	Rebif 44	20	2	27	2.08
	Betaferon	20	11	27	3.31
	Glatiramer acetate	30	8	40	2.45

General guidance regarding how to search for and select studies to be included in an NMA can be found in [22] and will not be further discussed in this manuscript.

#### Network diagram

**Fig. 1:**
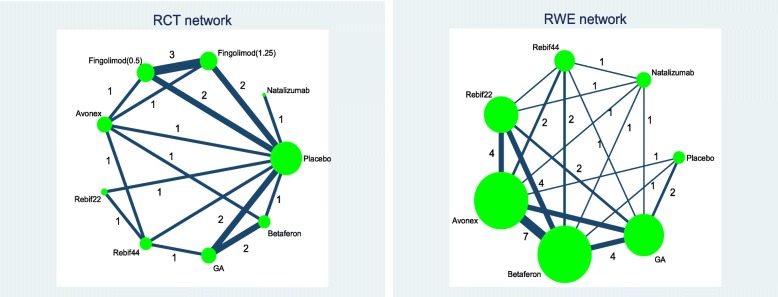
*Left panel*: network diagram of RCTs. *Right panel*: network diagram of RWE studies

**Fig. 2:**
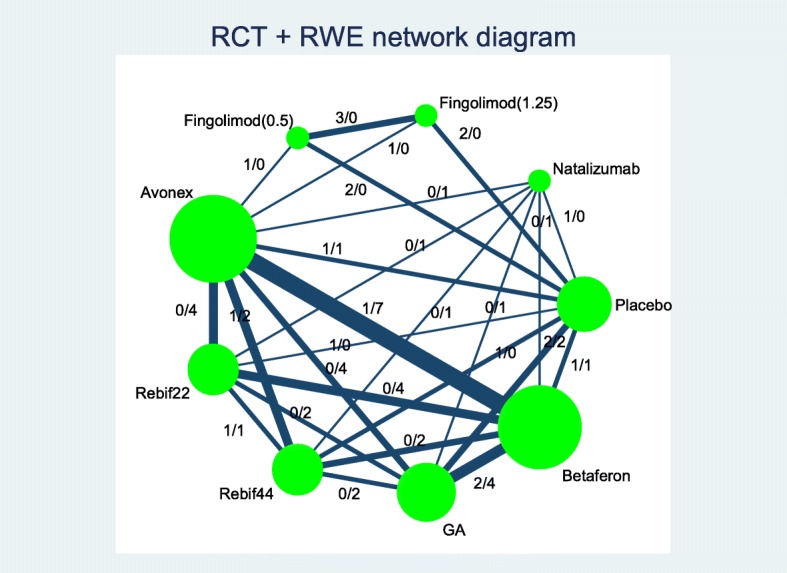
Network diagram including both RCTs and RWE studies up to the HTA submissions for fingolimod

Figure 1 shows the network diagrams including all RCTs (including the phase II trial) and RWE studies separately. Figure 2 shows the integrated network conducted up to the time of health technology assessment (HTA) submissions for fingolimod. There were 23 studies included in the NMA. Fourteen of these studies were RCTs and 9 studies were from RWE sources.

### Network-meta analysis methods

A random effects NMA that allows for variation of treatment effects across studies [23] was undertaken to synthesise data from both sources of evidence: the RCT data and RWE. The NMA was applied to both datasets individually and then to the two sources of evidence combined. In the combined meta-analysis model RWE was included at face value without weighting [24]. No weights or other adjustments were applied to reduce the impact of RWE data. To account for the different sources of evidence, a Bayesian hierarchical model was used [25].

Let *Y*_*ik*_ be the number of relapses in treatment *k* of study *i*. Then assuming a negative binomial (NBin) distribution, we have:

$$ {Y}_{ik}\sim NBin\left({\gamma}_{ik},{p}_{ik}\right) $$

where *p*_*ik*_is the probability of a relapse in treatment *k* of study *i* and *γ*_*ik*_ is the rate at which events (relapses) occur in arm *k* for study *i*.

For further details of the NMA models, including sensitivity analyses, the investigation of fixed and random effects models, the prior distributions on model parameters and the uniform prior distribution (UN[0,2]) of the between-subject standard deviation, we refer to Jenkins et al. [26].

**Fig. 3:**
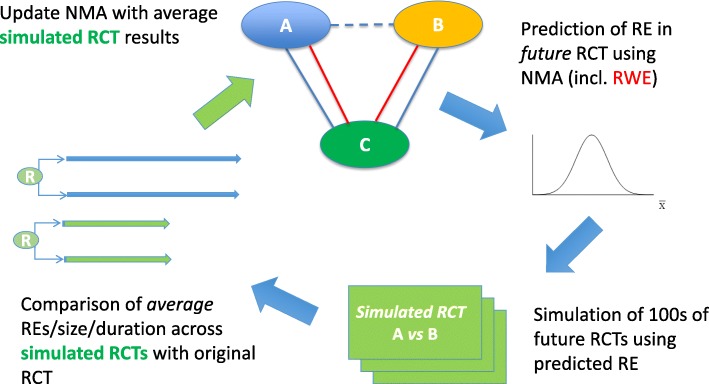
Graphical illustration of inclusion of RWE in the clinical development strategy

Based on the RCTs and RWE studies that have been published in RRMS, NMAs were performed at different stages of the development, after phase II of fingolimod [17] (Step 1) and after each pivotal trial in the development programme. A final NMA was also performed to include all data from RCTs and RWE studies in RRMS available prior to the HTA approval of fingolimod. The results of the NMA were used to simulate alternative phase III studies (Step 2) to investigate whether the early and strategic use of RWE could alter clinical decision making and help design more efficient clinical studies, resulting in lower patient numbers required. Figure 3 provides a graphical illustration of how the NMA was used to inform subsequent clinical trial simulations that were performed to illustrate the alternative development strategies.

Starting at the top of Fig. 3, assume there is a network of treatments A, B and C, with direct comparisons for A vs B and B vs C from RCT as well as from RWE data (red lines), and indirect comparisons for treatment A vs treatment B (dotted line in Fig. 3). Following Fig. 3 clockwise, the NMA including RWE can be used to predict relative effectiveness in an RCT. The obtained prediction estimates can be used to simulate alterative trials that compare, say treatment A vs treatment B (green boxes at the bottom of Fig. 3). The simulated effects, variability and duration of the trials can be compared with the results from the original clinical trials. Finally, the NMA can be updated with the simulated RCT results (green arrow in Fig. 3) to investigate the possible impact of future trials on estimates of effect in an NMA. Note that upon completion of the future trial, this can be added to the network instead of the simulated trial to see whether the results of the NMA were as predicted. This process can be repeated as new trials and information become available.

### Clinical trial simulations

The second step in the proposed strategy is to use the estimates from the NMA to simulate the effects and confidence interval of a future alternative phase III trial and assess how many patients will be needed to execute a phase III trial with sufficient power (90%), whilst the probability of falsely claiming effectiveness remains low (< 5%).

The estimate of the ARR and corresponding standard error obtained from the NMA which included all trials conducted up to the phase II trial of fingolimod [17], but before the execution of the (next) phase III trial, TRANSFORMS [18], FREEDOMS [20] or FREEDOMS II [21] were used to simulate the next phase III trial of fingolimod.

For a series of sample sizes (*n*_1_, *n*_2_,…), 1000 random samples were drawn from a negative binomial distribution to represent 1000 clinical trials of size *n*_*k*_. So for each trial, *n*_*k*_ samples were drawn from the negative binomial distribution (*Y*~ NBin(*r*, *p*)), where the random variable *Y* is the number of “failures” (no occurrence of a relapse) before the *r*^th^ “success”, in this case, the occurrence of a relapse. This procedure was repeated 1000 times.

Following the repeated sampling from the negative binomial distribution, a negative binomial regression was then carried out to compare treatments for each of the 1000 (simulated) clinical trials.

A general negative binomial model is defined by:

$$ \log {y}_k={\beta}_0+{\beta}_1{x}_{k1}+{\epsilon}_k={\mu}_k+{\epsilon}_k $$

where *y*_*k*_ is a random variable with a negative binomial distribution if we assume that the distribution of ${\tau}_k={e}^{\epsilon_k}$ has a gamma distribution [27] and *x*_*k*1_ is the indicator variable for treatment arm *k*. The NMA was performed on aggregate data, and as a result, no covariates were included in the negative binomial regression to minimise bias [28]. If individual patient data (IPD) are available, then the covariates known to have an impact on the response parameter can be included.

In Step 3 each trial was then analysed to investigate the number of patients needed to execute that trial, the power of the trial, i.e. *Probability*(reject the null hypothesis H_0_|H_1_ is true) and the probability of falsely claiming effectiveness, i.e. *Probability*(reject the null hypothesis H_0_|H_0_ is true), i.e. the false positive rate of that trial. The trials were analysed using a negative binomial regression model. For set levels of power the numbers of patients required were estimated. Similarly, for varying levels of patients, including the number of patients needed in the original trials, we estimated the number of times (out of 1000) that the null hypothesis was rejected when it is assumed that the “true” state is that treatments were superior to placebo (power) and the number of times (out of 1000) that the null hypothesis was rejected when in fact the “true” state is that the treatments were not superior to placebo (false positive rate).

These results were compared with those from the original trials.

This process can then be repeated at each key stage of development, i.e. before the start of the next (pivotal) phase III trial. Data on relapses were simulated from the results of the NMA. Based on the results of the NMA, patients of an alternative TRANSFORMS trial were simulated and analysed using a negative binomial regression model as described above. Alternative phase III trials were then simulated, and the number of patients needed to execute these simulated trials was evaluated. Subsequently, the published results of the ARR and 95% confidence intervals were then used to simulate whether or not the TRANSFORMS could have been executed with fewer patients (Step 4). Steps 2, 3 and 4 are repeated after each completed pivotal trial in the programme. In the last step (Step 5), the size and relative effect estimates of simulated trials instead of the original trial results were included in an NMA to investigate whether or not there is a difference in the totality of evidence of effectiveness between the original NMA and the NMA based on simulated results.

Following the clinical trial simulations, the sample sizes of the original studies and recruitment time were contrasted to those from the simulated studies. Based on the reported recruitment in the reported studies, the average recruitment rate (number of patients per month) was calculated and applied to the simulated scenarios. The recruitment time of the alternative strategies assumed this recruitment rate, and the time to recruitment was estimated accordingly.

Plots of treatment rankings were produced to compare the rankings of the original synthesised data and the new (simulated) alternative studies to investigate whether the treatment rankings were altered based on the proposed strategy.

### Software

The NMAs were performed in WinBUGS [29]. The clinical trial simulations were performed in the R programming language [30]. The graphical displays were created using STATA (network diagrams) [31], R (power curves) [30] and Microsoft Projects (for the figure of recruitment time; see Fig. 7 in the subsection titled Results of clinical trial simulations).

## Results

### Results of NMA

Including RWE studies not only increased the evidence base, but also the number of treatment comparisons not considered within the RCTs (see also Figs. 1 and 2). The phase II trial provided additional evidence for the comparison between fingolimod 1.25 mg and placebo.

Table 3 displays the ARR ratios (and standard errors) from an NMA of all RCTs (above the diagonal) and those obtained from the NMA of the RCTs and RWE combined (below the diagonal).

**Table 3:** Matrix table of annualised relapse rate ratios (standard errors) for an NMA of all RCTs (above the diagonal) and those obtained from the NMA of the RCTs and RWE combined (below the illustrated diagonal). Results presented at face value with no adjustments made for the inclusion of RWE

	Placebo	Nataluzimab	Fingolimod 1.25	Fingolimod 0.5	Avonex	Rebif 22	Rebif 44	Copaxone	Betaferon
Placebo		0.314 (0.03)	0.462 (0.03)	0.423 (0.03)	0.832 (0.06)	0.727 (0.07)	0.679 (0.05)	0.659 (0.04)	0.670 (0.05)
Nataluzimab	0.407 (0.07)		1.488 (0.20)	1.361 (0.20)	2.677 (0.30)	2.336 (0.30)	2.183 (0.30)	2.120 (0.30)	2.157 (0.3)
Fingolimod 1.25	0.455 (0.05)	1.150 (0.23)		0.918 (0.08)	1.808 (0.17)	1.581 (0.19)	1.476 (0.15)	1.433(0.14)	1.458 (0.15)
Fingolimod 0.5	0.413 (0.05)	1.045 (0.22)	0.916 (0.12)		1.977 (0.19)	1.728 (0.21)	1.614 (0.17	1.567 (0.16)	1.594 (0.17)
Avonex	0.783 (0.07)	1.977 (0.36)	1.742 (0.24)	1.920 (0.27)		0.877 (0.09)	0.818 (0.06)	0.795 (0.07)	0.808 (0.07)
Rebif 22	0.766 (0.08)	1.933 (0.36)	1.706 (0.26)	1.880 (0.30)	0.982 (0.10)		0.939 (0.08)	0.913 (0.10)	0.929 (0.10)
Rebif 44	0.7482 (0.08)	1.887 (0.35)	1.666 (0.26)	1.837 (0.30)	0.959 (0.10)	0.983 (0.11)		0.974 (0.07)	0.991 (0.09
Copaxone	0.601 (0.05)	1.517 (0.28)	1.338 (0.19)	1.474 (0.21)	0.771 (0.07)	0.790 (0.09)	0.809 (0.09)		1.019 (0.07)
Betaferon	0.700 (0.07)	1.768 (0.32)	1.559 (0.22)	1.718 (0.25)	0.897 (0.07)	0.920 (0.09)	0.943 (0.1)	1.170 (0.11)	

The ARR can be interpreted as the mean number of relapses per year. The ratio is the average relapse rate in the experimental arm compared to the control; e.g. the rate ratio of fingolimod (1.25 mg) over placebo is approximately 0.46, indicating that a patient on fingolimod has a 54% lower relapse rate compared to a patient on placebo.

The results of the NMAs that included all trials including the phase II trial of fingolimod were used to investigate the alternative clinical development strategies as described in the following section.

### Results of clinical trial simulations

**Fig. 4:**
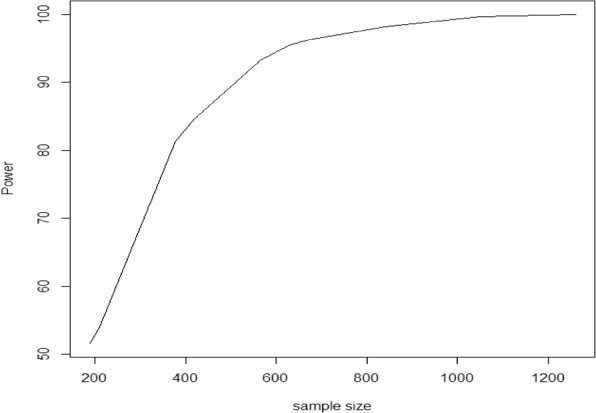
Power curve of the simulated alternative TRANFORMS study (1000 simulations)

Figure 4 shows the power curve of the simulated TRANSFORMS trial. The treatment effects used to simulate the power of an alternative TRANSFORMS trial were obtained from the NMA (RCT and RWE) that included the phase II trial. The figure shows that the power of the simulated TRANSFORMS trial exceeded 90% at 284 patients per arm (568 in total for a two-arm trial). The original TRANSFORMS trial was designed to have 90% power and recruited approximately 420 patients per arm (840 in total for a two-arm trial). The clinical trial simulations showed that the probability of falsely claiming superiority of the experimental drug vs standard of care was less than 5%. Note that the chosen significance level in the design of the TRANSFORMS trial was 5% [18].

The reduced sample size implies a potential savings of 30% of the originally planned sample size if all available evidence (including RWE) is used to design the trial.

Following the clinical trial simulations using RWE in conjunction with the phase II trial of fingolimod to design an alternative TRANSFORMS trial, the published results of the TRANSFORMS trial were analysed assuming that 284 patients per arm were recruited. Clinical trial simulations showed that the probability of observing effects (thus superiority of active over comparator) as reported in the TRANSFORMS trial was 85%. This simulated power was only marginally lower than the prespecified power (90%) that was used during the design stage of the executed TRANSFORMS trial. However, the 85% probability of observing a statistically significant difference compared to standard treatment could be achieved with 30% fewer patients as discussed previously.

**Fig. 5:**
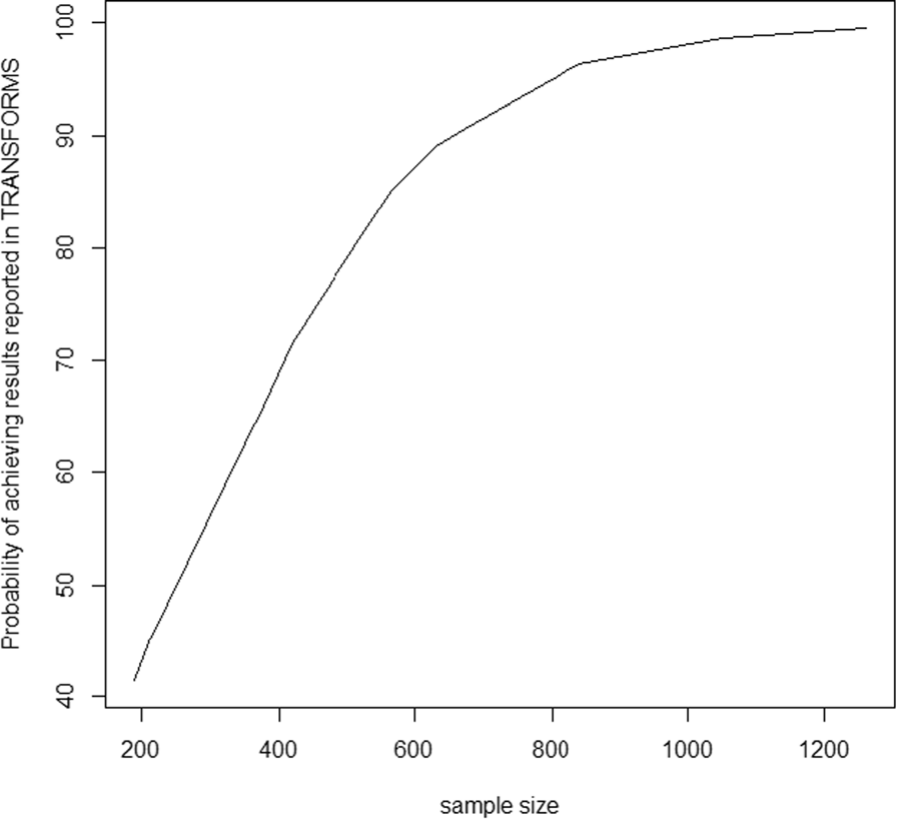
Probability of achieving results observed in the original TRANSFORMS study for varying sample sizes

Figure 5 outlines the probability of achieving superiority of experimental drug vs standard of care, assuming the results reported in Cohen et al. [18]. The curve illustrates that, assuming the effects reported, 90% success probability could be achieved with fewer patients, approximately 700 patients in total compared to the 840 patients originally recruited in two arms, a reduction of 17%.

**Fig. 6:**
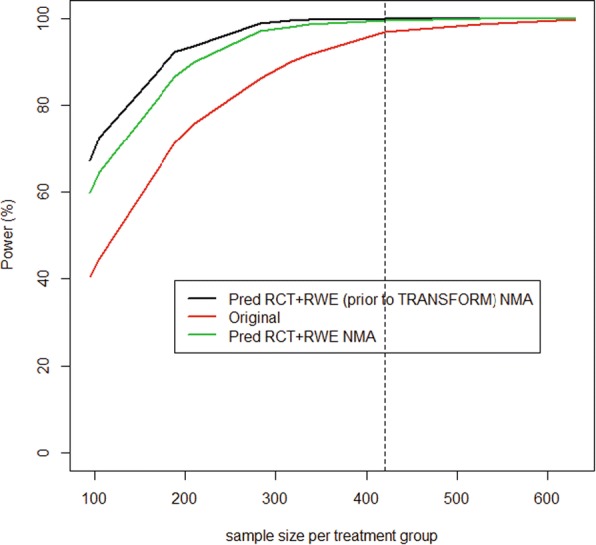
Power curves based on 1000 trial simulations of a trial of active vs comparator

Figure 6 shows the associated power curve for the trial simulations of the simulated TRANSFORMS trial using the effects from the NMA that included RWE prior to TRANSFORMS (black line), the power curve for the TRANSFORMS trial (assuming the published effects are observed, red line) and the power curve using the effects from the NMA that included all available data (green line). This demonstrates that, based on the original design, if RWE were to be included in the development programme, this could result in a significant savings in terms of patient numbers, recruitment time and ultimately costs.

In the alternative strategies, using a predicted trial effect based on an NMA of RCTs and the RWE studies that were available at the time of the design of the TRANSFORMS trial, the power is higher due to the relatively higher effects obtained from all the data in comparison to the increased uncertainty.

Similar evaluations were performed using the FREEDOMS and FREEDOMS II trials. Moreover, it was found that both the FREEDOMS and FREEDOMS II trials could be performed with 189 patients per group if RWE could be considered during the design stage (see alternative III below). This is plausible, since these studies were placebo controlled and not active controlled as the TRANFORMS trial, so fewer patients would be required.

**Fig. 7:**
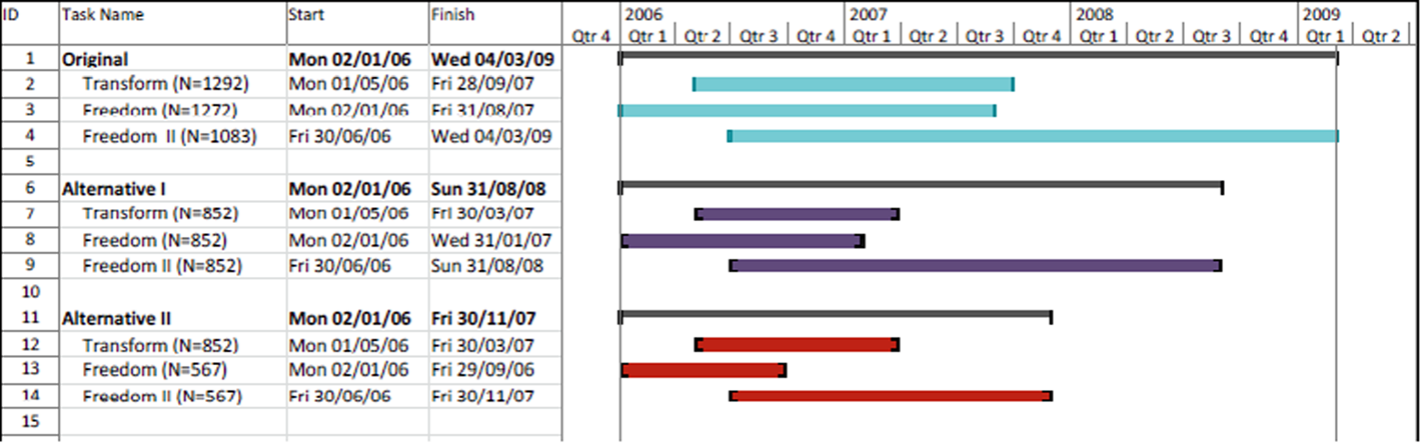
Recruitment times in original TRANSFORMS and projected recruitment times in simulated TRANSFORMS for two alternatives

Figure 7 shows the recruitment time of the original TRANSFORMS, FREEDOMS and FREEDOMS II trials and two alternatives based on alternative development strategies.

The following alternative strategies can be considered:

- *Option I (corresponding to alternative I* Fig. 7*)*. This option illustrates the original strategy to execute the studies with a similar number of patients. Using RWE to design the TRANSFORMS, the subsequent studies were assumed to require a similar number of patients. The timelines show that using RWE to design the clinical programme could result in a savings of 6 or 7 months on each trial.
- *Option II (corresponding to alternative II in* Fig. 7*)*. Clinical trial simulations of the FREEDOMS trials showed that the FREEDOMS II trial required a similar number of patients as the FREEDOMS trial (567 in total). In this option, the resulting savings would be remarkable: 6 months for TRANSFORMS, 11 months for FREEDOMS and 16 months for FREEDOMS II.
- *Option III (combining alternatives I and II).* Based on the number of patients needed to execute the alternative FREEDOMS II trial (mainly a North American population), the FREEDOMS II trial could be executed as a cohort of the FREEDOMS trial. This implies that the FREEDOMS II trial would not be required. The single trial would have more than sufficient power to analyse the patients recruited in FREEDOMS and the North American cohort consisting of 189 patients per arm (567 in total). The combined FREEDOMS trials could require a total of 1134 patients (2 × 567), and the FREEDOMS II trial would not be required as a separate trial, whilst the alternative FREEDOMS trial would still be marginally smaller than the reported trial with 1272 patients randomised.

Table 4 shows the results from the NMA which included the results of the simulated TRANSFORMS, FREEDOMS and FREEDOMS II trials. Note that the original TRANSFORMS, FREEDOMS and FREEDOMS II results were replaced with the simulated results in this NMA.

**Table 4:** Matrix table of annualised relapse rate ratios (standard errors) for an NMA of all RCTs, including the simulated TRANFORMS and FREEDOMS studies (above the diagonal) and those obtained from the NMA of the simulated RCTs and RWE combined (below the diagonal). Results presented at face value with no adjustments made for the inclusion of RWE

	Placebo	Nataluzimab	Fingolimod 1.25	Fingolimod 0.5	Avonex	Rebif 22	Rebif 44	Copaxone	Betaferon
Placebo		0.319 (0.05)	0.519 (0.05)	0.465 (0.05)	0.916 (0.09)	0.783 (0.10)	0.706 (0.07)	0.667 (0.06)	0.686 (0.07)
Nataluzimab	0.420 (0.08)		1.666 (0.31)	1.492 (0.28)	2.940 (0.54)	2.370 (0.54)	2.265 (0.42)	2.140 (0.39)	2.157 (0.41)
Fingolimod 1.25	0.497 (0.07)	1.210 (0.28)		0.901 (0.09)	1.781 (0.22)	1.437 (0.25)	1.373 (0.20)	1.297 (0.18)	1.335 (0.19)
Fingolimod 0.5	0.448 (0.07)	1.103 (0.26)	0.913 (0.13)		1.989 (0.25)	1.606 (0.28)	1.534 (0.22)	1.450 (0.20)	1.491 (0.22)
Avonex	0.831 (0.08)	2.039 (0.40)	1.703 (0.27)	1.886 (0.30)		0.811 (0.13)	0.774 (0.09)	0.732 (0.09)	0.753 (0.09)
Rebif 22	0.798 (0.10)	1.957 (0.40)	1.639 (0.30)	1.814 (0.33)	0.965 (0.11)		0.920 (0.14)	0.920 (0.14)	0.945 (0.16)
Rebif 44	0.781 (0.10)	1.914 (0.38)	1.604 (0.29)	1.776 (0.32)	0.944 (0.11)	0.987 (0.13)		0.951 (0.10)	0.980 (0.13)
Copaxone	0.614 (0.06)	1.507 (0.30)	1.261 (0.21)	1.396 (0.23)	0.743 (0.08)	0.777 (0.10)	0.794 (0.10)		1.033 (0.10)
Betaferon	0.728 (0.08)	1.788 (0.35)	1.496 (0.25)	1.656 (0.28)	0.881 (0.08)	0.921 (0.10)	0.941 (0.11)	1.193 (0.12)	

**Fig. 8:**
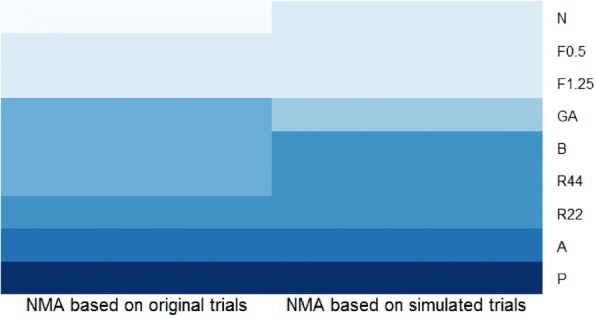
Heatmap of NMA based on original trials (*left*) and simulated trials (*right*)

The ARR and SE clearly show that the results of the NMA that included the simulated studies were very similar to those from the NMA that included the original trials, with only marginal increases in uncertainty despite the comparatively larger reduction in sample size (see also Table 3). The NMA based on the simulated trials did not alter the treatment rankings seen in previous analyses (See Fig. 8), indicating that the (simulated) smaller studies would not alter the overall evidence of effect. The lower ranked treatments in dark blue (starting from placebo (P)) from the NMA that included the original trials remained low in rank (dark blue) in the NMA that included the simulated trials. Similarly, the higher ranked treatments (light blue) remained higher in rank in both NMAs. The colour code of the intermediate treatments also remained similar in both analyses, indicating no change in their rankings between both NMAs.

Based on these results, one can conclude that in this example the use of RWE at key stages of development could result in smaller clinical trials, limiting the exposure of patients to an inferior treatment, without loss of evidence of effectiveness (see also Table 2). This strategy may result in an earlier regulatory and HTA approval and can contribute to addressing the medical needs of patients and patient groups.

## Discussion

The results from NMAs were used to inform the design of clinical trials in an example clinical development programme. Power curves were created through clinical trial simulations, assuming different scenarios of including RWE in the NMA. There were a number of advantages associated with this approach. We have demonstrated that the efficiency of clinical trial design can be improved (expressed in terms of lower patient numbers in a development plan of studies) by using RCT and RWE data together. We have also shown that inclusion of RWE in the clinical development strategy planning could result in a more efficient clinical development plan compared to having the development strategy be based on a single RCT. Note that the sample size calculations for the phase III studies were based on a Wilcoxon rank sum test, a non-parametric method, whilst our sample size estimations were based on clinical trial simulations using a negative binomial distribution, which is the underlying assumption in the method used for analysing the clinical trial data [18, 20, 21]. As a result, it is unclear what proportion of gain is due to the inclusion of RWE or the fact that the original sample size was based on a Wilcoxon rank sum test. The Wilcoxon rank sum test might have been more conservative in estimating the sample size, leading to the inclusion of more patients, compared to a sample size based on a negative binomial assumption which would correspond to the method used to analyse the data. In general, if distributional assumptions are met, a parametric approach for sample size estimation and data analysis is more powerful than a non-parametric approach [32, 33].

Although we have shown a positive effect (smaller phase III studies) of including RWE data in the development strategy, note that there may be instances when the inclusion of RWE may result in a larger trial in the programme due to increased heterogeneity between studies [8]. This does not necessarily imply that RWE should not have been used, but it may reflect the possibility that the effects observed in an RCT that did not include RWE are not representative of a target population under consideration and a larger trial incorporating knowledge from RWE patients may be more informative.

The RCT data are taken at face value without weighting to illustrate the use of an NMA for clinical trial design purposes. However, more elaborate analyses could be undertaken to weight the RCT and RWE data for rigour, bias and relevance [34]. Moreover, the analyses performed did not account for covariates, because only aggregate RCT and RWE data were available. In the presence of individual patient data, these analyses could be performed accounting for patient baseline characteristics [35]. The use of RWE for sample size calculation is an extension to the use of pairwise meta-analysis to design future trials [6–9]. Fewer patients per study may be needed and fewer pivotal studies required due to the totality of evidence included in the development strategy. As a result, an informed decision regarding effectiveness can be taken earlier, resulting in reduction of cost of clinical development, fewer patients being exposed and ultimately earlier accessibility to effective drugs or alternatively, when the evidence shows lack of effect, early termination of a drug that is not shown to be effective based on the totality of evidence included in the decision-making process. RWE can also provide evidence of effectiveness in the real world not measured in an RCT [36]. The NMA is recommended to be performed at key milestones, such as at the end of the phase II trial and prior to the design of key phase III studies, and repeated when new evidence becomes available. In short, it should be an integral part of the clinical development programme process. We adopted a Bayesian approach to undertaking the NMAs, as this naturally allows predications to be made which are crucial input to the trial simulation process [19]. Alternative approaches may also be considered in conjunction with the proposed strategy to include RWE, such as interim analyses that may include sample size re-estimation approaches, choice of alternative comparators or more flexible (adaptive) clinical trial designs. However, some of the pitfalls of these approaches may include increased sample size [37]. Kairalla et al. [38] described cases where appropriately designed adaptive trials could lead to reduced sample size and an increased chance of answering the clinical questions of interest. Kairalla et al. [38] also highlighted areas, such as comparative effectiveness studies, that may benefit from an adaptive design approach. Whatever approach is considered, care should be given to maximise the use of all available evidence and optimise the use of the patient data collected. The proposed strategy could increase the efficiency of flexible designs even further.

## Conclusion

The use of RWE resulted in a reduced sample size of the pivotal phase III studies, which lead to substantial time savings compared to the approach of sample size calculations without RWE. However, further case studies and simulation studies are required to assess the situations when such an approach may be particularly attractive.

## Abbreviations

ARR: Annualised relapse rate
EDSS: Expanded Disability Status Scale
HTA: Health technology assessment
NBIN: Negative binomial
NMA: Network meta-analysis
RCT: Randomised controlled clinical trial
RRMS: Relapsing remitting multiple sclerosis
RWD: Real world data
RWE: Real world evidence
